# CellSeg: a robust, pre-trained nucleus segmentation and pixel quantification software for highly multiplexed fluorescence images

**DOI:** 10.1186/s12859-022-04570-9

**Published:** 2022-01-18

**Authors:** Michael Y. Lee, Jacob S. Bedia, Salil S. Bhate, Graham L. Barlow, Darci Phillips, Wendy J. Fantl, Garry P. Nolan, Christian M. Schürch

**Affiliations:** 1 Department of Microbiology and Immunology, Stanford University School of Medicine, Stanford, CA 94305 USA; 2 Department of Pathology, Stanford University School of Medicine, Stanford, CA 94305 USA; 3 Department of Computer Science, Stanford, CA 94305 USA; 4 Department of Urology, Stanford University School of Medicine, Stanford, CA 94305 USA; 5 Department of Bioengineering, Stanford University School of Medicine, Stanford, CA 94305 USA; 6 Department of Dermatology, Stanford University School of Medicine, Stanford, CA 94305 USA; 7 Stanford Cancer Institute, Stanford University School of Medicine, Stanford, CA 94305 USA; 8 Department of Obstetrics and Gynecology, Stanford University School of Medicine, Stanford, CA 94305 USA; 9 Department of Pathology and Neuropathology, University Hospital and Comprehensive Cancer Center Tübingen, Tübingen, Germany

**Keywords:** Deep learning, Segmentation, Image analysis, CODEX, Mask R-CNN, Multiplexed imaging, Pre-trained model

## Abstract

**Background:**

Algorithmic cellular segmentation is an essential step for the quantitative analysis of highly multiplexed tissue images. Current segmentation pipelines often require manual dataset annotation and additional training, significant parameter tuning, or a sophisticated understanding of programming to adapt the software to the researcher’s need. Here, we present CellSeg, an open-source, pre-trained nucleus segmentation and signal quantification software based on the Mask region-convolutional neural network (R-CNN) architecture. CellSeg is accessible to users with a wide range of programming skills.

**Results:**

CellSeg performs at the level of top segmentation algorithms in the 2018 Kaggle Data Challenge both qualitatively and quantitatively and generalizes well to a diverse set of multiplexed imaged cancer tissues compared to established state-of-the-art segmentation algorithms. Automated segmentation post-processing steps in the CellSeg pipeline improve the resolution of immune cell populations for downstream single-cell analysis. Finally, an application of CellSeg to a highly multiplexed colorectal cancer dataset acquired on the CO-Detection by indEXing (CODEX) platform demonstrates that CellSeg can be integrated into a multiplexed tissue imaging pipeline and lead to accurate identification of validated cell populations.

**Conclusion:**

CellSeg is a robust cell segmentation software for analyzing highly multiplexed tissue images, accessible to biology researchers of any programming skill level.

## Introduction

Tissue imaging and single-cell analysis can reveal previously undetected biological structure and uncover subtle spatial relationships between cells. Recently, the development of antibody-based multiplexed imaging methods has enabled deep single-cell phenotyping of tissue microenvironments [1–9]. This analysis has been especially useful in cancer studies, where these imaging platforms have revealed nuanced tumor architecture and interactions between tumor, immune and stromal cells, and healthy host tissue [10–16]. In such highly multiplexed tissue imaging studies, the quality and accuracy of downstream analyses depend critically on the precise identification and correct phenotypic assignment of single cells, which requires accurate demarcation of each cell’s boundary and quantification of its marker expression. This is usually accomplished using an automated segmentation and signal quantification algorithm [17]. At a minimum, a segmentation algorithm takes an image as input and produces a set of masks denoting the boundary of each identified cell.

Commonly used segmentation algorithms include watershed (WTS) combined with thresholding [18, 19] and level-set techniques [20]. For example, WTS segmentation was recently used to identify cells in highly multiplexed fluorescence microscopy datasets of mouse and human tissues [3, 15, 16, 21]. However, these methods can be sensitive to noise within the image including blurred cell–cell contact boundaries and imaging artifacts such as antibody aggregates. Additionally, they are often not robust to variations in cell size or morphology and require significant parameter tuning for expected cell size, shape of nucleus, and cell density [22]. These limitations make their application to segmenting images of tumor tissue challenging, since cancers consist of a variety of cell shapes, sizes, and densities. Advances in deep learning architectures have transformed cell image analysis [23], and these models have been extended to applications in single-cell segmentation [24–31]. Leading among these algorithms is the Mask R-CNN architecture, which has previously shown positive performance on other segmentation tasks [26, 32]. However, deep learning algorithms usually require pre-labeled training data for the specific segmentation task, leading to substantial and time-consuming human input to obtain a high-quality segmentation.

While pre-trained deep learning architectures exist, such as StarDist [33] and Cellpose [26], an additional issue is the subsequent handling of the segmentation output to produce single-cell statistics used for downstream analysis, including pixel quantification. This results in either extra coding or exporting of masks to another image processing software where additional commands allow quantification of pixels in the segmented image to produce single-cell statistics. Further processing steps, including expanding mask boundaries and reducing noise in the statistics must be completed separately. Complete segmentation pipelines including CellProfiler [34] or ilastik [35] address these concerns but still often require hands-on user input, such as manual annotation or processing of segmentation results.

Here, we present CellSeg (https://michaellee1.github.io/CellSegSite/index.html), an easy-to-use, pre-trained, Mask R-CNN-based cell segmentation and pixel quantification software. Users supply a set of tissue images to CellSeg, and the software returns the segmented images and a table of single-cell statistics including each cell’s location, nucleus size, and mean pixel values in each imaging channel. CellSeg is open-source and available for Windows, Mac, or Linux. It is capable of segmenting JPG, PNG, and TIFF images of any image size, subject to hardware requirements reported below. CellSeg is accessible to individuals of all programming levels and requires minimal user input. For most uses, the pre-trained CellSeg model requires no additional manual annotation of training data, no additional training, and limited parameter tuning to produce a high-quality segmentation. The software has been designed to work as a library, so more advanced Python users can customize the pipeline to fit their needs. Applications detailed below demonstrate that CellSeg robustly segments highly multiplexed fluorescence images from a variety of healthy and cancerous tissues. CellSeg exceeds an established WTS segmentation pipeline both in terms of user friendliness as well as segmentation accuracy and performs at the level of two state-of-the-art deep learning segmentation algorithms.

## Implementation

### Overview of CellSeg pipeline

**Fig. 1:**
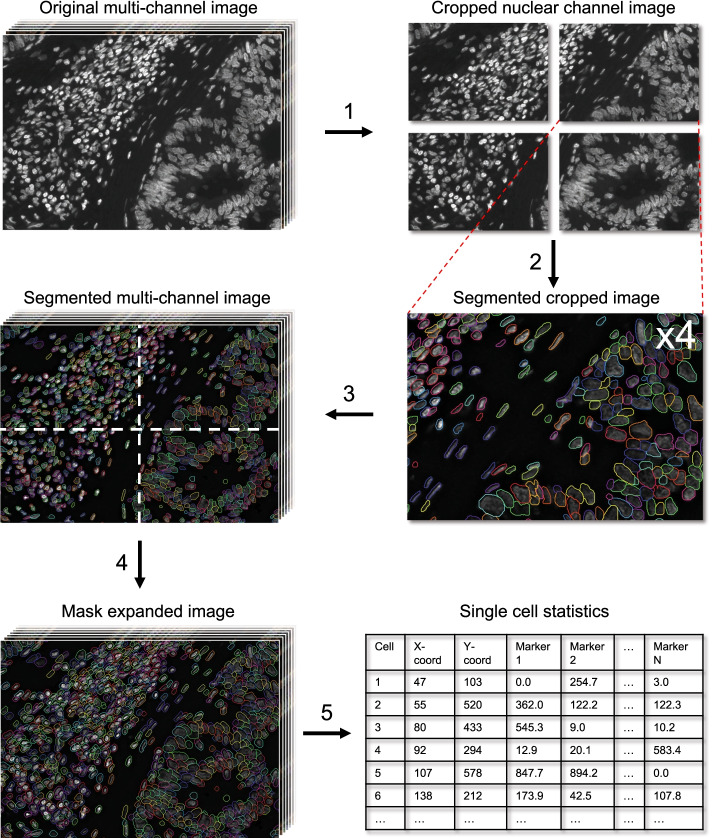
CellSeg pipeline. Overview of CellSeg software with following steps. (1) Extract nuclear channel and crop images to segment. (2) Segment each image crop with CellSeg. (3) Stitch together segmented crops. (4) Expand boundaries of cells using mask expansion. (5) Perform lateral bleed compensation, then compute and output single-cell statistics for N markers

The CellSeg software is implemented in Python and run using Jupyter Notebook [36]. CellSeg first extracts a user-specified nucleus color channel for segmentation (Fig. 1, step 1). Through iterative visual inspection, we found increasing the brightness of the nuclear channel can improve segmentation performance, especially in images with weak nuclear stain signal. CellSeg therefore scales each nuclear image’s brightness by a fixed constant computed from a reference image specified by the user. After scaling, CellSeg splits the nuclear stain image into several overlapping cropped images to accelerate segmentation (Fig. 1, step 1). Each image crop is segmented, and the resulting segmented crops are stitched into the full segmented multi-channel image (Fig. 1, steps 2–3). To eliminate erroneously segmented imaging artifacts, which often appear as high intensity speckles or clusters, CellSeg removes objects smaller than a user-specified threshold from the set of segmented cells.

After segmentation, two optional post-processing steps follow: mask expansion and lateral bleed compensation (Fig. 1, steps 4–5). Mask expansion extends the boundary of each segmented nucleus by a user-specified number of pixels to capture cell membrane fluorescent signal (Fig. 1, step 4). Lateral bleed compensation aims to correct fluorescent signal spillover between adjacent cells, an issue often seen in immunofluorescence imaging of dense tissues. The details of these steps and their performance are discussed below. After post-processing, CellSeg computes the mean pixel value for each marker over the set of pixels contained in each identified cell. In fluorescent images, these values can be seen as a proxy to the expression level of each imaged protein in each cell. CellSeg saves each cell’s (X, Y) coordinate and pixel quantifications to a table in both comma-separated value (CSV) and flow-cytometry standard (FCS) formats (Fig. 1, step 5). This data output format is recognizable by popular downstream single-cell analysis software, including flow cytometry gating programs such as CellEngine (https://cellengine.com), Cytobank (https://www.cytobank.org), or FlowJo (https://www.flowjo.com), as well as cell clustering programs like VorteX [37]. For visual inspection of segmentation quality, the user can also optionally generate images of the segmented tissue with overlaid masks and a TIFF stack of mask regions of interest (ROIs) which can be viewed in other image analysis programs like Fiji/ImageJ [38].

### User accessibility

CellSeg is an open-source segmentation software accessible to individuals with a range of programming skills. On the CellSeg webpage (https://michaellee1.github.io/CellSegSite/index.html), we created detailed tutorials for software installation, setup, and segmentation configuration. These tutorials assume no prior programming knowledge, and they walk the user through all phases of the CellSeg pipeline. The user has the option to run CellSeg using either a Jupyter Notebook containing a step-by-step walkthrough of the pipeline with comments or a fully automated script for segmenting several images. For more advanced users, the components of the pipeline can be used as a library. This allows users to incorporate algorithms from CellSeg into their personal segmentation pipelines or use CellSeg algorithms individually during exploratory image analysis. CellSeg can be run in the background on any computer with sufficient storage for the input image files with at least 16 GB RAM. While it does not require a GPU, CellSeg can be accelerated for those with access to hardware using the open-source package tensorflow-gpu [39], and instructions for GPU acceleration are provided on the webpage.

### Training

**Fig. 2:**
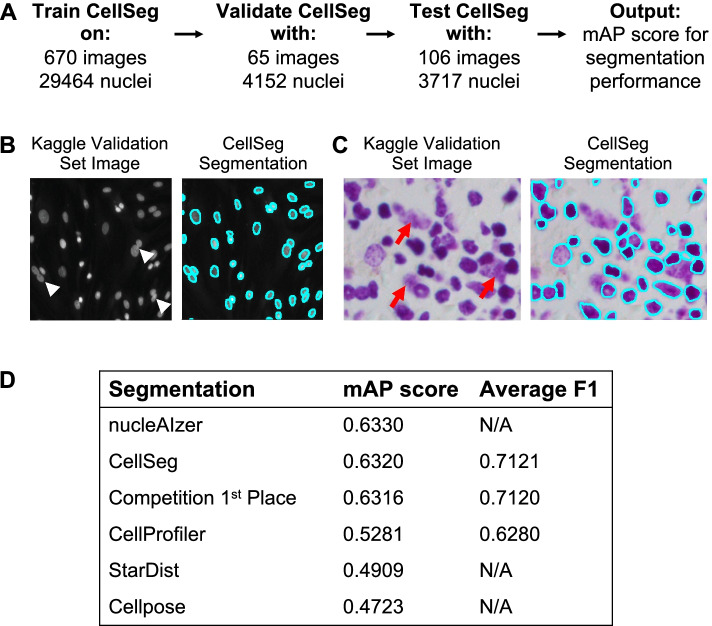
Training and Benchmarking CellSeg performance on the 2018 Kaggle data challenge. **A** Information on Kaggle dataset used to develop, train, and test CellSeg. CellSeg final performance was assessed on a test set provided by the Kaggle data challenge using mean average precision (mAP) score. **B** CellSeg segmentation of representative fluorescence image from the Kaggle test set. White arrowheads: cells with blurred nuclear boundaries **C** CellSeg segmentation of representative H&E-stained brightfield image. Red arrows: nuclear debris. **D** CellSeg performance compared to other top performing segmentation algorithms in data science bowl. Columns show mean average precision (mean AP) scores reported on Kaggle DSB2018 stage 2 test set and average F1 scores. For nucleAIzer, reported scores from the original publication [29] are displayed. For StarDist, brightfield and fluorescence images were segmented using 2D_versatile_he pre-trained model and 2D_versatile_fluo pre-trained model, respectively. For Cellpose, the pre-trained nuclei segmentation model was used (see “Methods” section for testing details)

CellSeg was trained on a dataset of fluorescent and brightfield biological microscopy images from the 2018 Kaggle Data Science Bowl containing 29,464 ground truth segmented nuclei (Fig. 2A) [40]. These images were acquired with variations in cell phenotype, size, image zoom, and brightness. Of note, CellSeg was not trained on any highly multiplexed tissue imaging data. Both the CellSeg architecture and training method were optimized for robustness to variations in cell size and morphology. Further details of CellSeg's architecture and training can be found in the “Methods” section.

## Results and discussion

### CellSeg architecture achieves high performance on Kaggle data challenge test set

After implementing CellSeg, we validated each step of the pipeline: architecture, segmentation, segmentation post-processing, and output. First, we quantitatively evaluated our trained Mask R-CNN segmentation architecture using the 2018 Kaggle Data Science Bowl [40]. This challenge dataset enabled assessment of the performance of our trained Mask R-CNN architecture in comparison with top performers in this competition and more recently published neural network-based cell segmentation algorithms such as nucleAIzer [29], StarDist [33], and Cellpose [26].

CellSeg was tested for segmentation quality on a ground truth segmented validation set of 3717 nuclei from Kaggle (Fig. 2A). We found that CellSeg qualitatively performed well on fluorescence images where it accurately identified individual nuclei even when boundaries between two nuclei were blurred (Fig. 2B). CellSeg accurately identified many nuclei in the brightfield image stained with hematoxylin & eosin (H&E) (Fig. 2C), but overall performed better in fluorescent images, due potentially to the higher amounts of nuclear debris and other artifacts in the H&E-stained images. The Kaggle challenge used mean average precision (mAP) as the segmentation quality metric (“Methods” section). A higher mAP score corresponds to a more accurate segmentation on the Kaggle competition’s test set. By the mAP metric, CellSeg performed among the top algorithms in the competition and comparably to nucleAIzer, while attaining a higher mAP score than both StarDist and Cellpose (Fig. 2D).

### CellSeg outperforms an established WTS segmentation algorithm on a multi-tissue microarray

**Fig. 3:**
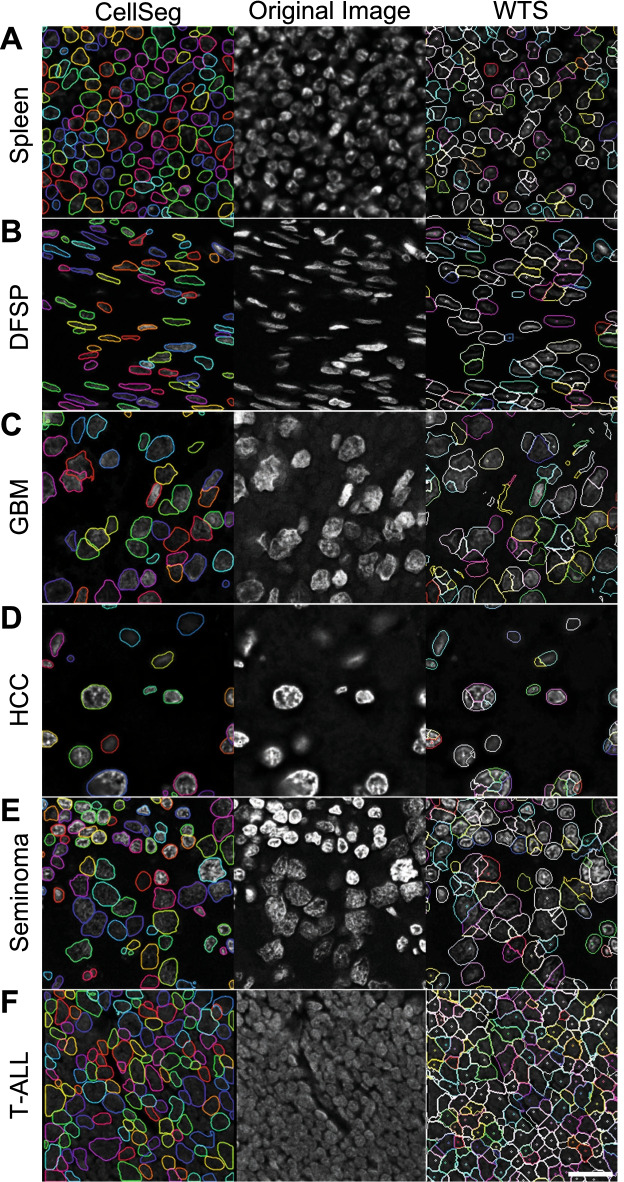
CellSeg performance on diverse human FFPE tissues. CellSeg performance on representative tissue images from a multi-tumor tissue microarray imaged with CODEX, all stains are DRAQ5 nuclear stain. **A.** Healthy spleen shows small cells. **B.** Dermatofibrosarcoma protuberans (DFSP) shows spindly nuclei. **C.** Glioblastoma multiforme (GBM) shows large, misshapen cells. **D.** Hepatocellular carcinoma (HCC) shows large, round cells. **E.** Seminoma shows a blend of large tumor cell nuclei and small nuclei from tumor-infiltrating lymphocytes. **F.** T-cell acute lymphoblastic leukemia (T-ALL) shows densely packed cells. Scale bar, 20 μm. Fluorescence intensity increased in original images for visualization purposes

Next, we evaluated CellSeg’s performance on a set of tissues representing a variety of cell and nuclear sizes, densities, and morphologies. Using a tissue microarray (TMA) comprised of human tumor and healthy tissues and imaged with the fluorescent nuclear marker DRAQ5 [15], we qualitatively assessed the performance of CellSeg against an established WTS algorithm that was tuned using hyperparameters selected by an expert pathologist for optimal segmentation quality [3]. We found that CellSeg was less sensitive to variations in nucleus size and morphology, outperforming the WTS algorithm on most tissues (Fig. 3). Both CellSeg and WTS correctly segmented immune cells and cells with spindly nuclei (Fig. 3A, B). While WTS tended to incorrectly segment large nuclei into several smaller masks in glioblastoma multiforme (GBM), hepatocellular carcinoma (HCC), and seminoma tissues, CellSeg correctly identified both large and small nuclei in the same images (Fig. 3C–E). CellSeg robustly identified nuclei with significant variations in brightness, as observed in the GBM and HCC tissues. CellSeg and WTS with expert annotation did not perform well on an image of T-cell acute lymphoblastic leukemia (T-ALL), a highly dense tumor composed of small lymphocytes with obscured nuclear boundaries (Fig. 3F). Manually segmenting such tissues is challenging even for expert pathologists. These findings underscore the importance of both tissue quality and clear separation between individual cells as key parameters for optimal segmentation. Overall, CellSeg performed at least as well as the established WTS algorithm on all tissues, while clearly outperforming it on three tissues (GBM, HCC, and seminoma).

### CellSeg performs at the level of state-of-the-art neural network-based segmentation algorithms

**Fig. 4:**
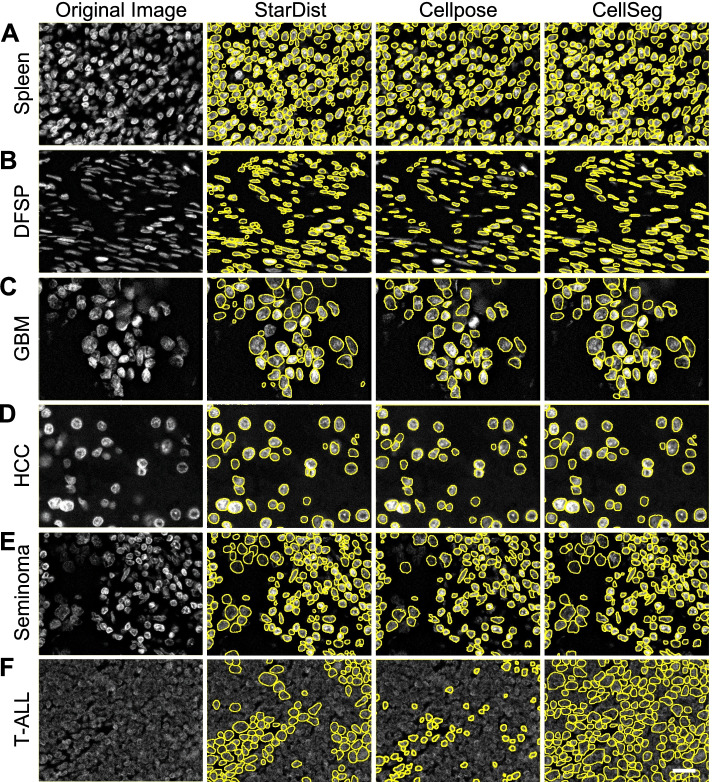
CellSeg performs comparably to established deep learning-based segmentation algorithms on diverse human FFPE tissues**.** Representative images from tissues described in Fig. 3 are shown. **A.** StarDist, Cellpose, and CellSeg show comparable performance on spleen. **B.** StarDist oversegments several spindly nuclei in DFSP (arrows), while CellSeg and Cellpose segment nuclei accurately. **C.** StarDist and CellSeg segment more low intensity objects in GBM (arrows). **D.** all three algorithms perform similarly well on HCC. **E.** StarDist and CellSeg segment more low intensity objects in seminoma (arrows). **F.** All three algorithms perform relatively poorly on T-ALL. Scale bar, 20 μm

Next, we qualitatively compared CellSeg’s performance to two recently published neural network-based segmentation algorithms, StarDist [33] and Cellpose [26]. The six tissues from our TMA shown in Fig. 3 were segmented using pre-trained models of StarDist (2D versatile fluo) and Cellpose, and the segmentation masks were compared to masks generated by CellSeg. All three algorithms performed comparably well on spleen and HCC (Fig. 4A, D). Compared to CellSeg, StarDist segmented more objects with low fluorescence intensity in GBM and seminoma (Fig. 4C, E). However, StarDist also oversegmented many nuclei in DFSP, while CellSeg identified them accurately, suggesting that CellSeg is more robust to variations in nuclear morphology (Fig. 4B). Both StarDist and CellSeg identified more nuclei than Cellpose in DFSP, GBM, and seminoma (Fig. 4B, C, E). All three algorithms performed poorly on T-ALL (Fig. 4F). In summary, CellSeg performs at the level of state-of-the-art neural network-based segmentation algorithms when applied to a real-world dataset.

### CellSeg post-processing steps improve downstream resolution of immune populations

**Fig. 5:**
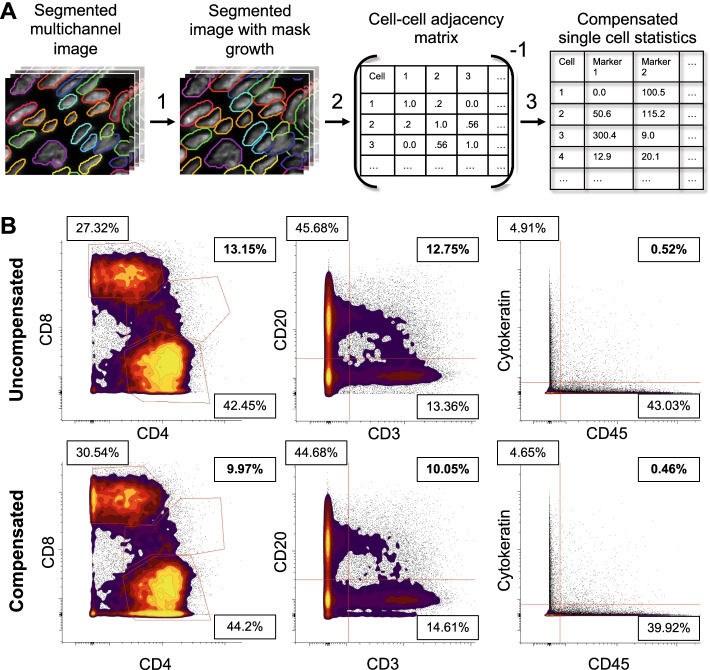
Testing lateral bleed compensation on a CODEX dataset of colorectal cancer samples. **A.** Schematic demonstrating post-processing of CellSeg segmentation with following steps. (1) Grow cell boundaries by user defined number of pixels (growth of two pixels shown). (2) Compute inverse adjacency matrix from cell–cell. (3) Multiply inverted adjacency matrix by marker pixel intensity vector to obtain compensated single-cell pixel quantifications table. **B.** Effects of lateral bleed compensation on double-positive cell populations in the CRC dataset for three pairs of mutually exclusive markers (CD8 vs. CD4, Cytokeratin vs. CD45, CD20 vs. CD3). Data shown are from one of the two CRC TMAs (TMA A), with comparable bleed compensation performance for the other TMA (TMA B, data not shown)

In the development of CellSeg, we addressed two post-segmentation issues. First, because CellSeg is a nucleus segmentation algorithm, the boundary identifying a cell’s nucleus often fails to capture the fluorescent signal of its plasma membrane where many of the protein markers used for cellular identification are located (e.g., CD45 denoting an immune cell, and EpCAM denoting an epithelial cell). Second, fluorescent imaging often results in spatial fluorescent spillover between adjacent cells, creating noise in quantification of protein expression [3]. To resolve these issues, two optional steps follow segmentation with CellSeg: (1) mask expansion (Fig. 5A, step 1) and (2) lateral bleed compensation (Fig. 5A, steps 2–3). Mask expansion extends the boundary surrounding each segmented nucleus by a user-defined number of pixels. This allows for quantification of the plasma membrane fluorescent signal. Next, lateral bleed compensation corrects for fluorescence spillover between adjacent cells. As described in Goltsev et al. [3], this algorithm computes the surface contact ratios between physically adjacent cells and uses this value to simultaneously boost signal from a cell and reduce spatial spillover noise from neighboring cells.

We tested the efficacy of the lateral bleed compensation algorithm with CellSeg on an immunofluorescence dataset imaged on the CO-Detection by indEXing (CODEX) platform. CODEX iteratively visualizes protein-antibody binding events, allowing for the quantification of more than 50 protein targets in formalin-fixed, paraffin-embedded (FFPE) or fresh-frozen tissue sections [3, 15]. Using CODEX, we recently imaged two TMAs containing 140 samples from 35 patients with colorectal cancer (CRC). In this study, WTS was used to segment the images [15]. This particular WTS segmentation used the same bleed compensation algorithm that we implemented for CellSeg. Single-cell marker quantifications from the WTS segmentation were extensively validated in this study, providing us with a baseline for the expected expression profiles of cell types against which we could validate our bleed compensation algorithm.

Using CellSeg, we segmented the 140 CRC images either with or without bleed compensation and gated the segmented data in CellEngine. In this dataset, the expression of certain pairs of imaged protein markers are expected to be mutually exclusive based on their known biology, including CD20/CD3, CD8/CD4, and cytokeratin/CD45. However, the presence of several densely populated immune cell regions, as observed in the CRC dataset, can lead to the erroneous identification of cells that are positive for both markers due to spatial fluorescent spillover. Applying an approach previously used to assess compensation [3], we measured the efficacy of bleed compensation by the observed reduction in the frequency of cells double positive for any of these pairs of markers (Fig. 5B). Lateral bleed compensation reduced the frequency of double positive cells in both the CD8/CD4 and CD20/CD3 pairs, with a lesser reduction of double positive cells in the cytokeratin/CD45 pair. Therefore, the lateral bleed compensation implemented in CellSeg improves the resolution of immune cell expression profiles in a dataset with densely packed immune cell regions.

### Phenotyping using CellSeg output recapitulates validated cell populations in the CRC CODEX Dataset

In previously published work using the CODEX pipeline, WTS segmentation provided single-cell fluorescent intensity statistics that were used to assign cell phenotypes in the CRC dataset [15]. To assess whether CellSeg could replace WTS in the CODEX pipeline, we performed a head-to-head comparison by gating segmented cells in all 140 samples with key phenotyping markers of major cell types as validated by an expert pathologist.

**Fig. 6:**
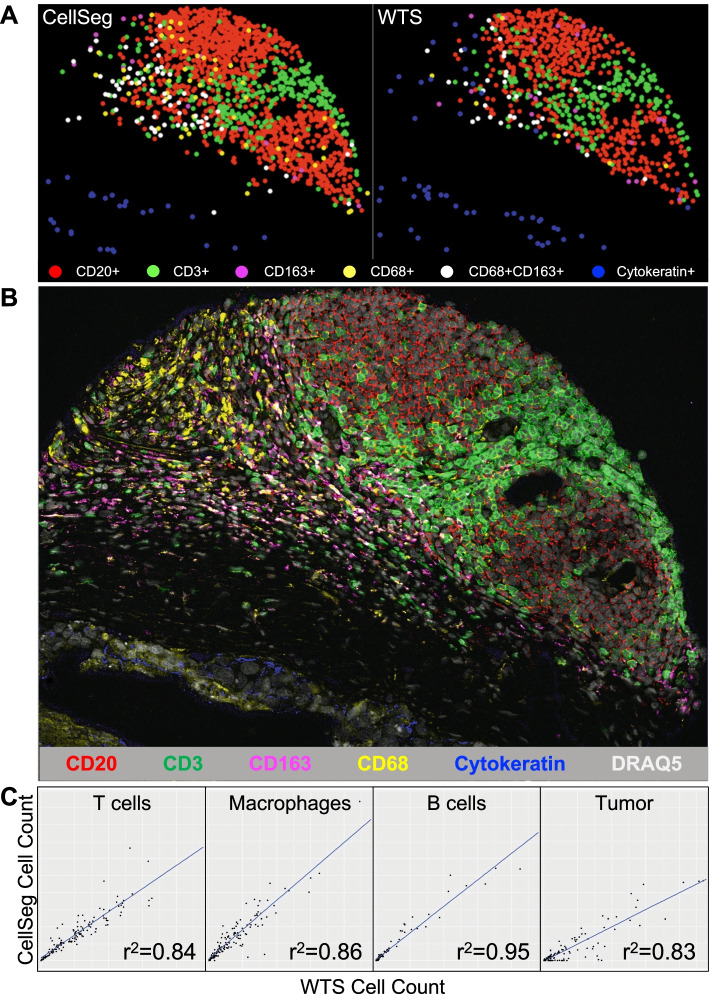
Recapitulating previously identified cell populations using CellSeg. **A.** Visualization of identified populations in a representative CRC tissue. Points on scatter plots show positions of cells on the displayed tissue image in Fig. 5B. Population identity obtained through gating. **B.** Fluorescent image of a representative CRC tissue. Expression of six phenotyping markers used in Fig. 5A shown. **C.** Population correlation analysis between CellSeg and WTS. Each point corresponds to a TMA spot, where the X value is the gated population count from WTS and the Y value is the count computed from CellSeg. Least-squares regression line displayed along with r^2^ value. T cells are defined as CD45^+^CD3^+^CD20^−^Cytokeratin^−^; macrophages as CD45^+^CD20^−^CD3^−^ and CD68^+^, CD163^+^, or CD68^+^CD163^+^; B cells as CD45^+^CD20^+^CD3^−^Cytokeratin^−^; and tumor as Cytokeratin^+^CD45^−^

To confirm that the gated cell types derived from WTS and CellSeg occupied similar regions within the CRC tumors, we directly visualized these cell types on the CRC images. This analysis showed that CellSeg and WTS generated the same cell phenotypes with comparable spatial organization within the CRC tumors (Fig. 6A). Further validation was performed by examining the original fluorescent image displaying key phenotyping markers (Fig. 6B). Both CellSeg and WTS cell types, identified by gating, had the expected cell morphology and fluorescence marker profile when inspecting the corresponding regions of the fluorescent image.

For a more global quality assessment of our analysis, we correlated the cell types quantified by CellSeg with those identified by WTS. For each sample, we correlated the absolute number of cells in each gated population using the outputs of CellSeg and WTS (Fig. 6C). As examples, we depict gated T cells, macrophages, B cells, and tumor cells, all of which showed very strong positive correlations between the WTS and CellSeg segmented cell counts. The cell types generated from the CellSeg segmentation matched previously validated cell types generated from WTS in the CRC dataset. However, while cell counts from CellSeg and WTS were correlated, WTS segmentation generally resulted in higher numbers of tumor cells (Fig. 6C). This is likely due to over-segmentation of large tumor cell nuclei, as observed in GBM, HCC, and seminoma (Fig. 3C–E), suggesting that CellSeg is superior to WTS for tumor cell identification. These findings demonstrate that tissue analysis using CellSeg can recapitulate previously published findings in a multiplexed fluorescence imaging study.

## Conclusions

In summary, we present CellSeg, a robust single-cell segmentation and quantification software for tissue images. Our software has been designed to be accessible to researchers of all programming skill levels. For novice programmers, we have created detailed tutorials on how to implement and use CellSeg (https://michaellee1.github.io/CellSegSite/index.html). For more advanced Python users, the components of the CellSeg pipeline function as a library to complete customized image analysis or segmentation tasks. As more sophisticated segmentation algorithms emerge, future researchers can use the CellSeg pipeline and combine it with their algorithm of choice. Importantly, our pre-trained segmentation algorithm works “out-of-the-box” for many single-cell segmentation tasks, without requiring any additional manual training by the user.

We validated each step of the CellSeg pipeline: architecture, segmentation, post-processing, and output. In a post-competition evaluation, the pre-trained CellSeg architecture scored among the top performers from the 2018 Kaggle data challenge. CellSeg also qualitatively outperformed an established segmentation algorithm on a multi-tissue TMA. Both qualitative and quantitative comparisons demonstrated that CellSeg performs comparably to state-of-the-art deep learning-based segmentation algorithms. Finally, using a CODEX CRC dataset, we showed that the post-processing steps in our CellSeg pipeline improved resolution of mutually exclusive cell populations while recapitulating previously published cell populations in the dataset. CellSeg is therefore a powerful tool that has shown robust performance on a wide variety of tissue segmentation tasks and should help researchers with their needs in cell segmentation and marker quantification in biological imaging data.

## Methods

### Architecture

CellSeg is based on the Matterport implementation of Mask R-CNN [32, 41]. CellSeg uses a slightly modified loss function during training. The original Mask R-CNN paper uses L = L_cls_ + L_box_ + L_mask_, where the loss is the additive sum of class loss, bounding box loss, and mask loss as defined in the paper. A new hyperparameter was added to arrive at L = αL_cls_ + L_box_ + L_mask_. Through experimental analysis, it was found that reducing the contribution of class loss in our single-class model improved convergence during training. For the results in this paper, α = 0.5.

### Training

To prevent overfitting and to extend the training data, multiple image augmentation techniques were used, which contributed significantly to CellSeg’s quantitative performance. The first was simple random field sampling. At train time, 512 × 512 pixel crops of the image were used, selected randomly from the image. Using the imgaug library version 0.2.9, contrast normalization, brightness, Gaussian blur, zooms scaling the X and Y axes independently, vertical and horizontal flips, and rotations were also modulated throughout training [43]. No augmentations were conducted at test time. The model was trained in minibatch sizes of 16 using stochastic gradient descent with momentum. Transfer learning was utilized for the dataset, with weights used from a Mask R-CNN model that trained on COCO, a segmentation challenge with 91 object types and 2.5 million labeled instances [44]. Auxiliary functions for training were adapted from the DeepRetina DSB2018 training scripts, although our model did not use their trained weights [42]. The network was trained for 150 epochs with decaying learning rate with the base model frozen, to let only the top layers train. Then, the full network was trained for 25 epochs at a very low learning rate. All training was done on an Nvidia GTX 1080 Ti GPU and a Dual Intel® Xeon® Silver 4114 10-core CPU, taking about 41 h to train.

### Mean average precision metric

For each segmented image, mean average precision was computed using intersection over union (IoU) between segmented masks and ground truth masks as follows. IoU compares the overlap between the CellSeg segmentation of a cell and a ground truth manual segmentation. First, for each segmented mask, the pixel IoU, defined as the ratio of the overlap between ground truth mask A and CellSeg mask B to the total area that A and B cover was computed as IoU(A, B) = (A ∩ B)/(A ∪ B). IoU values range from 0 to 1, with 1 denoting a perfectly segmented cell, i.e., the ground truth. The number of cells with IoU values exceeding threshold t were computed, where t ranges from 0.5 to 0.95 in increments of 0.05. At each t, a precision value Q(t) was calculated as: Q(t) = TP(t)/(TP(t) + FP(t) + FN(t)) where TP, FP, and FN were the number of true positives, false positives, and false negatives identified in an image, respectively. A TP is defined as an object with a pixel IoU above the threshold t. The Average Precision (AP) of an image was then computed as the mean over the thresholds: AP = (1/|thresholds|)∑_t_Q(t). Finally, the mean AP (mAP) was computed as the mean over the AP of each image in the test dataset.

### Quantitative segmentation evaluation

Mask predictions were made on Kaggle DSB 2018 stage 2 test set images for CellSeg and uploaded to the DSB 2018 page on the Kaggle website (after the competition closed) to obtain mAP score. For StarDist (version 0.7.1), fluorescence and brightfield images were segmented using 2D_versatile fluo and 2D_versatile_HE pre-trained models, respectively. For Cellpose (version 0.6.5), fluorescence images were segmented using channels = [0,0] with all other parameters set to default. Brightfield images were segmented using parameters channels = [1,0], invert = True, and flow_threshold = 0.8 with all other parameters set to default. Predicted masks for each segmentation algorithm were saved and uploaded to Kaggle to obtain the mAP score.

### TMA qualitative segmentation evaluation

CellSeg and an optimized WTS segmentation algorithm [3] were both used to segment six tissue images from a multi-tissue TMA. Representative images from the results of each segmentation were selected for Fig. 3. We used the best-focus image returned by the 3D WTS algorithm to compare segmentation results. Size parametrization for WTS was hand-verified by a board-certified surgical pathologist (C.M.S.). For qualitative comparison between CellSeg, StarDist, and CellPose in Fig. 4, 300 × 400 pixel patches were sampled randomly from each tissue image to visualize. StarDist (version 0.7.1) 2D_versatile_fluo pre-trained model was used for segmentation with default parameters. For Cellpose, the pre-trained model (version 0.6.5) with no modifications was used. Segmentation results were visualized with mask ROI overlays in ImageJ.

### Mask expansion algorithms and lateral bleed compensation

We implemented two mask expansion algorithms. The first algorithm expands the boundary of each mask by a user-defined number of pixels. If this expansion leads to two overlapping masks, the algorithm assigns each pixel in the overlapping region to the mask whose center is closest to the pixel. The first mask expansion algorithm is computationally efficient and works well for tissue images with cells of similar size. However, the algorithm biases pixel assignment towards smaller masks, since the center of these masks are generally closer to the overlap region than the centers of larger masks. To correct for this, the second expansion algorithm iterates over the masks, expanding each mask by 1 pixel until it collides with another pre-existing mask boundary, at which point growth in that direction stops. The algorithm proceeds until each mask has been expanded by the user-defined number of pixels. This algorithm mitigates the need for assigning pixels based on distance to cell center at the cost of more computation time. Through iterative visual inspection of masks with and without mask expansion, we found that growth by 1 or 2 pixels is usually sufficient to capture most membrane protein signal. The lateral bleed compensation algorithm implemented in the CellSeg pipeline is the same as in previously published work from our group, readers are directed to the original paper for the details of the algorithm [3].

### Benchmarking CellSeg

Segmentation of the CRC dataset and multi-tissue TMA was performed on a Dual Intel® Xeon® Silver 4114 10-core CPU. Resulting segmented data was gated in CellEngine (https://cellengine.com). When evaluating fluorescent bleed compensation on the CRC dataset, samples were aggregated by TMA, resulting in two gates for each cell population, one corresponding to each TMA. When performing population correlation analysis, cell types were gated by sample, resulting in tailored gates for the 140 samples. Gating was performed independently for the WTS dataset and the CellSeg dataset in a blinded fashion (J.S.B.). Resulting identified populations were validated by an expert in flow cytometry (C.M.S.). Population correlation analysis was performed in R Studio. The script is available upon request. Plots in Fig. 6a and c were generated using the R package ggplot2 [45]. The tissue image for Fig. 6b was obtained in Fiji/ImageJ [38].

## Availability and requirements

Project Name: CellSeg. Project home page: https://michaellee1.github.io/CellSegSite/index.html. Operating system(s): Windows, MacOS, or Linux. Programming language: Python. Other requirements: Web browser, internet connection, Conda, Jupyter, minimum 16 GB RAM, other Python package dependencies listed on project home page. License: MIT. Restrictions for Non-academics: None.

## Data Availability

Raw data and materials used in this study are published and available for download [15, 40]. The gating data and associated statistics shown in Figs. 5 and 6 are available from the corresponding author on reasonable request.

## Abbreviations

CODEX: CO-detection by indexing
CPU: Central processing unit
CRC: Colorectal cancer
CSV: Comma-separated values
DFSP: Dermatofibrosarcoma protuberans
FCS: Flow cytometry standard
FFPE: Formalin-fixed paraffin-embedded
GBM: Glioblastoma multiforme
GPU: Graphics processing unit
HCC: Hepatocellular carcinoma
IoU: Intersection over union
mAP: Mean average precision
R-CNN: Region-convolutional neural network
ROI: Region of interest
T-ALL: T-cell acute lymphoblastic leukemia
TMA: Tissue microarray
WTS: Watershed
